# Supplementary material for: Perinatal exposure to ambient fine particle air pollution and risk of childhood ewing sarcoma in a population-based case-control study in California (1988–2015)
Source: Environ Health. 2025 Mar 7;24:6. doi: 10.1186/s12940-025-01159-6 (PMC11887085; doi:10.1186/s12940-025-01159-6)
Supplement: Supplementary file 1 — Supplementary Material 1 [file 12940_2025_1159_MOESM1_ESM.docx]

**Title**: Perinatal exposure to ambient fine particle air pollution and risk of childhood Ewing sarcoma in a population-based case-control study in California (1988-2015)

**Authors**: Cassandra J. Clark^1,2^, Rong Wang^3^, Joseph L. Wiemels^4^, Catherine Metayer^5^, Nicole C. Deziel^1,6^, Xiaomei Ma^3,6^

^1^ Department of Environmental Health Sciences, Yale School of Public Health, New Haven, Connecticut, United States

^2^ Department of Pediatrics, University of Minnesota School of Medicine, Minneapolis, Minnesota, United States

^3^ Department of Chronic Disease Epidemiology, Yale School of Public Health, New Haven, Connecticut, United States

^4^ Center for Genetic Epidemiology, Department of Population and Public Health Sciences, University of Southern California, Los Angeles, California, United States

^5^ Division of Epidemiology, School of Public Health, University of California, Berkeley, California, United States

^6^ Yale Comprehensive Cancer Center, Yale School of Medicine, New Haven, Connecticut, United States

**Corresponding Author:** Cassandra Clark (clar1168@umn.edu)

**Abstract**

**Background:** Incidence of childhood Ewing sarcoma, a rare cancer affecting bones and soft tissues, is increasing. Environmental exposures during the perinatal period, like air pollution, may play a role. We examined exposure to perinatal ambient fine particulate matter (PM_2.5_) and childhood Ewing sarcoma risk in a case-control linkage study nested within a California birth cohort.

**Methods**: The study included 388 children born in California (1982-2015) and diagnosed with Ewing sarcoma at age 0-19 years (1988-2015), and 19,341 California-born cancer-free controls frequency-matched to cases on birth year (50:1 ratio). Ambient PM_2.5_ concentrations at the maternal residence were averaged separately over two time periods, gestation and the first year after birth, using a validated ensemble-based model (categorized as quartiles). We estimated odds ratios (ORs) and 95% confidence intervals (CIs) for the association between perinatal PM_2.5_ exposure and Ewing sarcoma risk, adjusting for sex, birth year, race, ethnicity, birth weight, and maternal education and stratifying by Hispanic ethnicity to assess potential disparities in PM_2.5_-related cancer risk.

**Results**: In the overall population, perinatal ambient PM_2.5_ exposure was not associated with Ewing sarcoma risk when considering exposure during gestation or the year after birth. Among Hispanic children, who experienced greater air pollution exposure compared to non-Hispanic children, higher PM_2.5_ levels during gestation yielded elevated odds of Ewing sarcoma compared to the first quartile (Q2 OR [95% CI]=1.53 [0.94-2.51]; Q3=1.56 [0.95-2.56]; Q4=1.39 [0.79-2.47]). Hispanic children also experienced elevated risk in relation to exposure during the year after birth.
**Conclusion**: Our results provide new suggestive evidence that ambient PM_2.5_ may contribute to Ewing sarcoma risk, although these findings were not statistically significant and were specific to Hispanic children. These findings require replication and underscore the need to further evaluate the potential role of ethnicity in the PM_2.5_-cancer relationship with genetic ancestry measures and through the lens of environmental justice.**Introduction**

Little is known about the etiology of Ewing sarcoma. Ewing sarcoma is a rare, aggressive cancer of the bones and soft tissues occurring mainly in children and adolescents, and most commonly among males and Caucasians.^1^ While the prognosis for children in whom the disease is localized is fair (5-year survival: 65-80%), once metastasis occurs the survival rate drops dramatically (5-year survival: ~30%; survival with relapse: 10%).^2-5^ Further, survivors face increased risk of other health burdens like chronic illnesses (e.g., heart disease),^6-9^ psychological issues (e.g., depression, anxiety),^10^ and second primary cancers.^8, 11^ Though the peak incidence of Ewing sarcoma is between ages 10 and 15 years, the incidence of Ewing sarcoma has been increasing in younger children (0-9 years) in North America.^12^ The increasing incidence in younger age groups could suggest that prenatal or early life exposures may influence Ewing sarcoma risk.

The development of Ewing sarcoma generally results from balanced chromosomal translocations between the *EWS* gene and a member of the *ETS* family of genes.^2, 13, 14^ The most frequent translocation partner is *FLI-1*, resulting in an *EWS-FLI-1* fusion protein, which interferes with genetic transcription and RNA processing, thereby enhancing oncogenesis.^14-17^ Evidence from cell models suggests that the presence of *EWS-FLI-1* alone may not be sufficient to cause Ewing sarcoma, and that co-occurring mutations are necessary.^18-20^ Currently, understanding of the cell of origin for Ewing sarcoma is still limited,^14, 21, 22^ and the potential for environmental exposures to play a role in these events must be explored.

Existing knowledge on environmental risk factors for childhood Ewing sarcoma is scarce, with the largest body of evidence for pesticide exposure.^23-28^ Occupational parental exposures to pesticides and exposures during pregnancy have been linked to Ewing sarcoma risk, further underscoring a prenatal or early life origin.^25, 27^ Proximity to urban industrial activity has also been implicated, particularly to industries releasing air pollutants like polycyclic aromatic hydrocarbons.^29^ There have been a number of investigations of potential clusters of Ewing sarcoma that were hypothesized to have an environmental cause.^30-32^ Spatial clusters of the disease could indicate a role for spatially varying environmental risk factors, such as air pollution, in the development of Ewing sarcoma. Air pollution induces oxidative stress, inflammation, and oxidatively damaged DNA,^33-35^ which could provide a pathway for carcinogenesis. Outdoor air pollution is a known human carcinogen (IARC Group 1)^36^ and a known or suspected risk factor for childhood cancer.^37-40^ In particular, exposure to ambient PM_2.5_ (air pollution comprised of particles of aerodynamic equivalent diameter of 2.5 µM or less) has been linked to the risk of multiple types of childhood cancer, including lymphoid leukemia, retinoblastoma, Hodgkin and non-Hodgkin lymphomas, and central nervous system tumors (e.g., ^41-45^). The prenatal period in particular has been implicated as a critical window of exposure to ambient air pollution for some childhood cancers.^39,45^

While a growing body of evidence suggests a link between air pollution and other types of childhood cancer, this relationship has not yet been explored with regard to Ewing sarcoma risk. Moreover, marginalized racial and ethnic groups experience disproportionate exposure to air pollutants,^46-49^ as well as variability in background cancer incidence.^50^ In this study, we evaluated the relationship between perinatal exposure to PM_2.5_ and childhood Ewing sarcoma risk in a population-based case-control study nested within a California birth cohort, with a focus on identifying potential exposure and health outcome disparities among marginalized groups.

**Methods**

*Study Population and Data Sources*

This analysis leverages the California Linkage Study of Early-onset Cancers (CALSEC), a linkage of diagnoses of early-onset cancer (age 0-39 years) reported to the California Cancer Registry ^51^ from 1988-2015 and statewide birth records from 1982-2015. CALSEC also includes millions of controls who were born in California and were not diagnosed with any cancer at the age of 0-39 years based on information from the California Cancer Registry. The study protocol has been approved by the Institutional Review Boards at the California Health and Human Services Agency and Yale University.

Our source population included all 479 children who were born in California during 1982-2015 and diagnosed with a first primary malignant Ewing sarcoma (International Classification of Diseases for Oncology, 3^rd^ edition code 9260) at the age of 0-19 years during 1988-2015 (i.e., cases), as well as 23,950 control children frequency-matched to cases on year of birth at a 50:1 ratio. Matching on year of birth maintains the overall age distribution and can allow us to examine the potential impact of temporal variability in PM_2.5_ composition and concentrations.^52^ Control children were cross-checked with the cancer registry to ensure no prior diagnosis of cancer. Although CALSEC includes individuals diagnosed with cancer through age 39 years, we selected the age range of 0-19 years to examine potential environmental risk factors specifically for childhood and adolescent Ewing sarcoma, which has been underrepresented in the etiologic literature. We also elected to examine perinatal exposures specifically due to biological plausibility and that this window has been identified as a critical window of exposure for other childhood cancers, such as leukemia.^53^ As assessment of PM_2.5_ exposure is based on maternal residential address at birth, we removed cases and matched controls who had missing or inadequate (i.e., only zip code) data on maternal residential address. This yielded a final study population of 388 cases and 19,341 controls.

**Individual level data on demographic and birth characteristics (e.g., sex, race, ethnicity, birth weight, birth order) were abstracted directly from birth records. Socio-economic status (SES) is an important potential confounder in the relationship between air pollution exposure and health outcomes,^54^ and is difficult to characterize accurately.** In our study, we examined multiple representations of SES at both the individual- and community-level. We obtained individual-level maternal educational attainment from the birth record. For community-level measures of SES, we obtained census tract-level educational attainment and poverty statistics and the Centers for Disease Control/Agency for Toxic Substances and Disease Registry Social Vulnerability Index (SVI), a composite metric representing 15 different social, economic, and demographic domains.^55^ Each variable was evaluated for inclusion in the final model.

***PM_2.5_ Exposure Assessment***

We modeled daily outdoor PM_2.5_ concentrations at a spatial resolution of 1 km x 1 km using a validated ensemble model.^56^ The machine learning-based model combines random forest regression, a gradient boosting machine, and an artificial neural network and incorporates a wide variety of predictors, including satellite, land-use, meteorological, and chemical transport data.^56^ The estimates produced by this model have been found to be highly concordant with measured PM_2.5_ concentrations in the Pacific region (R^2^ = 0.802).^56, 57^ Daily concentrations were available from 2000-2016; for children born prior to 2000, PM_2.5_ was extrapolated using a regression model with year as a continuous variable and calendar month as a categorical variable. This approach has been used in a study of childhood leukemia.^58^ **Because little is known about potential critical windows of exposure for Ewing sarcoma, two perinatal exposure windows were considered: i) gestation to birth and ii) the first year after birth, both using the maternal residence at birth.^59, 60^ For each of the two time windows, monthly** PM_2.5_ concentrations **were averaged to produce a composite estimate. We evaluated PM_2.5_ categorically and continuously but elected to use the former for the final analyses due to evidence in the literature of a nonlinear relationship between PM_2.5_ exposure and other health outcomes (e.g., ^61, 62, 63^) particularly when PM_2.5_ concentrations are high.**

*Statistical Analysis*

All statistical analyses were conducted in SAS 9.4, and all tests were two-sided with an alpha level of 0.05. We used chi-square and t-tests to identify differences in the distribution of population characteristics between case and control children. To explore the complicated relationship between air pollution, SES variables, and cancer risk,^54^ we evaluated the distribution of these risk factors among different strata, such as Hispanic ethnicity and age at diagnosis. We conducted two primary regression analyses. In the first, we used unconditional logistic regression to estimate odds ratios (ORs) and 95% confidence intervals (CIs) for the association between PM_2.5_ exposure and Ewing sarcoma risk, adjusting for year of birth (the matching variable), sex, race, ethnicity, birth weight (continuous, per 500g), and maternal educational attainment, which were identified as risk factors in a previous analysis of this population.^64^ For the second, because people of color are more likely to be disproportionately exposed to environmental hazards including air pollution in the United States (US),^46, 47, 49, 65-74^ we **also stratified models by race and ethnicity to evaluate the influence of such exposure inequality. These exposure disparities could lead to disparities in risk, which could also be related to other social and structural factors.** Separate models were constructed for each window of exposure considered and all models included the same covariates as the primary models unless explicitly stated otherwise.

We also conducted sensitivity analyses to evaluate the robustness of our findings. **First**, differences in childhood cancer incidence have been noted among Hispanic children with foreign-born mothers as compared to US-born mothers (i.e., “the Hispanic epidemiologic paradox”),^75^ where children of foreign-born mothers exhibited incidences more similar to the maternal country of origin. To examine whether this phenomenon could influence risk patterns among Hispanic children, we conducted an additional analysis including whether the mother was foreign-born in the models. While an indication of a foreign-born mother does not necessarily represent a child’s genetic ancestry and it is critical not to conflate population descriptors with ancestry,^76^ it may provide an indicator of possible effect to be followed up on with more detailed ancestry information. Second, because birth weight may also be related to air pollution exposure and thus could feasibly be on the causal pathway,^64^ we conducted a sensitivity analysis excluding birth weight from the models.

**Results**

*Population Characteristics*

Case children were more likely to be male (57% vs. 51%, p=0.03) and be non-Hispanic White (46% vs. 34%, p<0.01). Case children had a significantly higher birth weight, on average (3434g vs. 3363g, p=0.02). Mothers of case children were somewhat more likely to have some college or higher-level educational attainment (34% vs. 29%, p=0.09), though this difference was not statistically significant. Case children tended to be born in areas of lower socio-economic vulnerability than control children (SVI percentile: 57.0 vs. 62.1, p<0.01); this pattern also held for domain-specific SVI metrics, including economic and minority status indices.

**Table 1. Characteristics of the study population.**

|  | Cases (n=388) | Controls (n=19341) | p-value |
| --- | --- | --- | --- |
| Sex | N (%) | N (%) | 0.03 |
| Male | 220 (57) | 9870 (51) |  |
| Female | 168 (43) | 9471 (49) |  |
|  |  |  |  |
| Age at diagnosis (yrs) |  |  | - |
| 0-4 | 50 (13) | 2464 (13) |  |
| 5-9 | 70 (18) | 3527 (18) |  |
| 10-14 | 143 (37) | 7133 (37) |  |
| 15-19 | 125 (32) | 6217 (32) |  |
|  |  |  |  |
|  |  |  |  |
| Gestational age (weeks) |  |  | 0.24 |
| 32 to <37 | 34 (9) | 1659 (9) |  |
| 37 to <39 | 84 (22) | 4163 (22) |  |
| 39-41 | 238 (61) | 11418 (59) |  |
| ≥42 | 31 (8) | 1780 (9) |  |
| Missing | 1 (1) | 321 (2) |  |
|  |  |  |  |
| Birth weight (grams) | Mean (SD*) | Mean (SD*) | 0.02 |
|  | 3434 (577) | 3363 (516) |  |
|  |  |  |  |
| Mode of delivery |  |  |  |
| Vaginal | 302 (78) | 14940 (77) | 0.78 |
| Cesarean | 86 (22) | 4401 (23) |  |
|  |  |  |  |
| Race and ethnicity |  |  | <0.01 |
| Non-Hispanic White | 177 (46) | 6484 (34) |  |
| Non-Hispanic Black | 1 (1) | 1500 (8) |  |
| Hispanic | 179 (46) | 9219 (48) |  |
| Asian/Pacific Islander | 29 (7) | 1951 (10) |  |
| Other | 2 (1) | 187 (1) |  |
|  |  |  |  |
| Mother’s educational attainment |  |  | 0.09 |
| 8th grade or less | 33 (9) | 2071 (11) |  |
| 9th - 12th grade | 130 (34) | 6797 (35) |  |
| Some college or more | 133 (34) | 5570 (29) |  |
| Unknown | 92 (24) | 4903 (25) |  |
|  |  |  |  |
|  | Mean (SD*) | Mean (SD*) | t-test p-value |
| Percent of block group with a college education or more | 47.2 (20.3) | 46.0 (20.9) | 0.26 |
| Percent of block group in poverty | 25.1 (18.4) | 27.3 (18.9) | 0.02 |
|  |  |  |  |
| Social Vulnerability Index percentile (total) | 57.0 (29.4) | 62.1 (27.7) | <0.01 |
| SVI socio-economic domain percentile | 55.5 (29.1) | 59.8 (28.1) | <0.01 |
| SVI Minority status and language percentile | 55.8 (29.7) | 60.6 (27.6) | <0.01 |

* SD: standard deviation

***PM_2.5_ Exposure***

Case and control children were exposed to similar ambient concentrations of PM_2.5_ at their birth residence across both time windows of exposure examined (Table 2). For example, in the gestational period, the average exposure among case children was 22.59 µg/m^3^ as compared to 23.27 µg/m^3^ among control children (t-test p-value = 0.29). However, when stratified by Hispanic ethnicity, several notable differences emerged (Supplemental Material, Table S2). In the combined population of cases and controls, Hispanic children were consistently exposed to significantly higher absolute (approximately 3-4 µg/m^3^) concentrations of PM_2.5_ than non-Hispanic White children across both exposure windows (p<0.01 for both windows). Hispanic case children were also exposed to significantly higher levels of PM_2.5_ across both exposure windows (all p<=0.01), with the difference being most pronounced in the gestational window (Hispanic case mean: 25.13 µg/m^3^ vs. non-Hispanic White case mean: 20.62 µg/m^3^; p<0.01). **Average exposure levels for all individuals decreased over time, with mean gestational exposure levels being roughly 10** µg/m^3^ **lower among children born in 2000 or later as compared to those born before (Supplemental Figure S1). However, exposure among Hispanic children was consistently higher than that of non-Hispanic White children across the duration of the study period.**

**Table 2. Associations between exposure to modeled average ambient PM_2.5_ concentrations during gestation and first year after birth and Ewing sarcoma risk.**

|  |  | |  |  |
| --- | --- | --- | --- | --- |
|  | Cases (n=388) | Controls (n=19341) | p-value | OR (95% CI)* |
| PM_2.5_ (µg/m^3^) |  |  |  |  |
|  | Mean (SD) | Mean (SD) |  |  |
| Gestational Period | 22.59 (12.37) | 23.27 (12.41) | 0.29 |  |
|  |  |  |  |  |
|  | N (%) | N (%) | 0.48 |  |
| Q1 (<13.68) | 105 (27) | 4836 (25) |  | 1.00 |
| Q2 (13.68 - <20.08) | 100 (26) | 4834 (25) |  | 0.98 (0.72, 1.32) |
| Q3 (20.08 - <29.90) | 99 (25) | 4836 (25) |  | 1.03 (0.76, 1.40) |
| Q4 (≥29.90) | 84 (22) | 4835 (25) |  | 0.87 (0.61, 1.25) |
|  |  |  |  |  |
|  | Mean (SD**) | Mean (SD**) |  |  |
| First Year after Birth | 21.82 (11.60) | 22.46 (11.70) | 0.28 |  |
|  |  |  |  |  |
|  | N (%) | N (%) | 0.70 |  |
| Q1 (<13.47) | 107 (28) | 4835 (25) |  | 1.00 |
| Q2 (13.47 - <19.48) | 92 (24) | 4835 (25) |  | 0.95 (0.70, 1.29) |
| Q3 (19.48 - <28.76) | 96 (25) | 4836 (25) |  | 1.09 (0.81, 1.49) |
| Q4 (≥28.76) | 93 (24) | 4835 (25) |  | 0.84 (0.58, 1.20) |
| *Adjusted for individual-level factors: birth year, sex, birth weight, race, ethnicity, and maternal educational attainment. ** SD: standard deviation. | | | | |

**
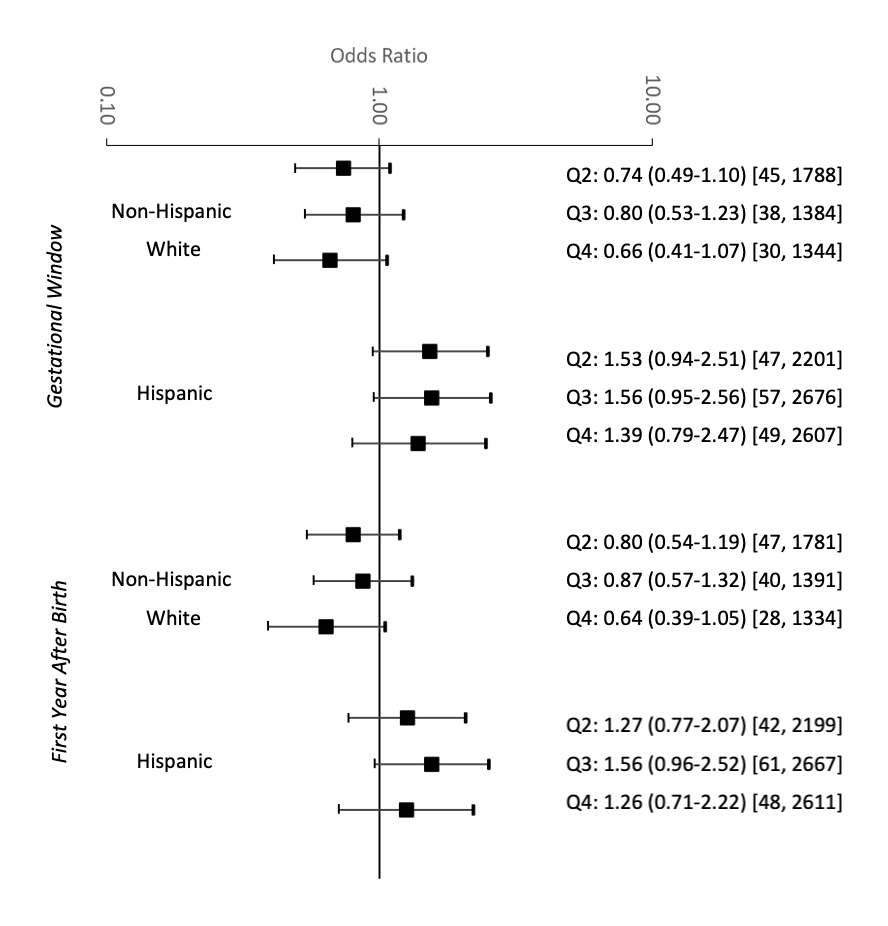
Figure 1. Associations between modeled PM_2.5_ concentrations and Ewing sarcoma risk, stratified by Hispanic ethnicity.**

Q = Quartile. [N cases, N controls]. All models adjusted for individual-level factors: birth year, sex, birth weight, and maternal educational attainment.

***PM_2.5_ Exposure and Childhood Ewing Sarcoma Risk***

In the overall population, there was no association between ambient perinatal PM_2.5_ exposure and Ewing sarcoma risk during gestation or the first year after birth (Table 2), adjusted for birth year, sex, birth weight, race, ethnicity, and maternal educational attainment. Stratifying by ethnicity revealed that exposure to PM_2.5_ during gestation and the first year after birth was associated with borderline elevated risk among Hispanic children, but not non-Hispanic White children (Figure 1). Hispanic children in the second and third quartiles of exposure during the gestational window had 1.53 (95% CI: 0.94-2.51) and 1.56 (95% CI: 0.95-2.56) times the odds of developing Ewing sarcoma, respectively, as compared to children in the lowest quartile of exposure. Similarly, we observed modestly elevated odds for Hispanic children in the third quartile of exposure during the first year after birth (OR: 1.56, 95% CI: 0.96-2.51). Adding an indicator of a foreign-born mother to the model did not substantially change any of the results (Supplemental Table S3). Removing birth weight did not substantially change any of the results (Supplemental Table S3).

**Discussion**

We examined the association between perinatal ambient PM_2.5_ exposure and childhood Ewing sarcoma risk in a large case-control study nested within a California birth cohort. In this study, we observed that **modeled PM_2.5_ concentrations during gestation and early life were not associated with Ewing sarcoma risk in the overall population. However, among Hispanic children, PM_2.5_ exposure during gestation or the first year after birth was associated with modestly elevated odds (30-60%) of developing Ewing sarcoma. These results add new evidence to a limited body of literature on environmental risk factors for the understudied childhood Ewing sarcoma and highlight disparities in both PM_2.5_ exposure and Ewing sarcoma risk.**

**We observed no association between PM_2.5_ exposure and Ewing sarcoma risk in the overall population. The potential environmental etiology of childhood Ewing sarcoma remains obscure. Animal models indicate a potential role for chemicals like benzophenone,** o-nitrotoluene, and riddelliine **in sarcoma development,^77, 78^ but not specifically Ewing sarcoma. In the human literature, much of the evidence for environmental risk factors for childhood Ewing sarcoma to date has focused on pesticides, including parental occupational exposures.**^23-27^ **Though multiple studies have reported elevated risk of malignant neoplasms including sarcomas associated with air pollution exposure,^32, 42, 79, 80^ there are few studies examining the influence of PM_2.5_ exposure on childhood Ewing sarcoma risk specifically. Williams et al. observed elevated Ewing sarcoma risk associated with PM exposure, though the confidence intervals were wide, perhaps due to a limited number of cases (n=47).^44^ Notably, the authors reported that 43% of the Ewing sarcoma cases included in the study lived in areas with the highest levels of PM_2.5_ observed in the study.^44^ It is important to note that PM_2.5_ is mixture that may be comprised of many different compounds and may vary geographically and seasonally.^52^ Therefore, replication of these studies in different populations and locations is critical to elucidate the true relationship between air pollution and childhood Ewing sarcoma risk.**

**Our study population experienced relatively high exposure to PM_2.5_ on average, often exceeding the health-based standards that existed during the study period. In 1997, the US Environmental Protection Agency enacted a health-based standard of 15.0** µg/m^3^ **for PM_2.5_. Most recently, in 2024, the Agency revisited the National Ambient Air Quality Standard for PM_2.5_, reducing the annual mean exposure limit from 12.0 (enacted in 2012) to 9.0** µg/m^3^**.^81^ In part due to the inclusion of children born as early as 1982, our study population had a relatively high mean exposure, exceeding the 1997 exposure limit by 7** µg/m^3^ **and exceeding the 2012 and 2024 exposure limits by more than 10** µg/m^3^**. Hispanic children in particular experienced persistently high levels of exposure across our study period, with the median exposure post-2000 exceeding both the 2012 and 2024 standards.**

**The exposure inequality we observed among Hispanic children in California likely resulted from multiple complex social and structural factors. We found that Hispanic children were consistently exposed to significantly higher levels of ambient PM_2.5_ than non-Hispanic White children. These results agree with other studies of air pollutants and cancer risk that reported Hispanic women and children living in California are exposed to higher burdens of air pollutants than non-Hispanic White individuals (e.g., ^48, 82, 83^); this pattern has also been reported extensively in North America more broadly (e.g., ^84, 85^). In addition to reaffirming this well-documented disparity in exposure, our results also indicated that perinatal PM_2.5_ exposure was associated with an elevated Ewing sarcoma risk of 30-60% among Hispanic children, but there was no association among non-Hispanic White children. This could indicate disparity in the exposure-response relationship among Hispanic children.**

**We explored the influence of multiple potentially explanatory factors for the elevated risk among Hispanic children despite the lower expected incidence overall.^64^ One potential hypothesis for the disparity seen among Hispanic children in our study is that children with foreign-born mothers could be experiencing differential risk as compared to those with domestic-born mothers. A comparison of childhood cancer risk among Hispanic children of US-born and non-US-born mothers reported some differential risk among childhood cancer subtypes; whether that risk was elevated or decreased as compared to non-Hispanic White children varied by subtype.^75^ In that study, Hispanic children of foreign-born mothers tended to exhibit similar cancer incidence patterns to those seen in the maternal birthplace. In our study, having a foreign-born mother did not appear to influence risk.**

**Another potential explanation for the differential risk among Hispanic children is variability in the relationship between air pollution exposure and socioeconomic status (SES, a number of social, economic, and lifestyle factors that can influence exposures and health outcomes; e.g., household income, poverty status) by ethnicity. Ewing sarcoma risk is thought to be associated with a higher SES. However, in our study, mothers of Hispanic children had lower levels of educational attainment than those of non-Hispanic children. While individuals with lower SES are more likely to be burdened with harmful environmental exposures,**^46, 47, 49, 65-74^ **there was still a trend of reduced risk of Ewing sarcoma among Hispanic children with mothers with lower educational attainment. Literature from other groups on this topic presents similarly mixed results. In a large multi-state study of multiple types of childhood cancer, lower maternal educational attainment was non-significantly associated with elevated childhood Ewing sarcoma risk.^86^ Further, in a study using data from the Surveillance, Epidemiology, and End Results program, sarcoma risk was found to be elevated in some racial and ethnic groups independently of census tract-level socioeconomic status.^87^ SES can be difficult to capture, and expected patterns (e.g., increasing educational attainment and its impacts on household income) may vary significantly between different racial and ethnic groups based upon differing social and structural pressures.^88^ SES may also vary by genetic ancestry, specifically within Latinx populations.^89^ Given the profound ancestry-related differences in risk, a gene-environment interaction study may help to explain these phenomena. Ultimately, in our study, adjusting for multiple individual- and community-level representations of SES did not attenuate the observed associations. However, we are constrained to information drawn from the birth record, and it is possible that there was some unaccounted for social or structural factor present among Hispanic or non-Hispanic children, which could include factors such as healthcare access and increased risk of other environmental exposures. Future studies may benefit from including more sophisticated individual-level measures of SES including household income.**

**This study has several notable strengths. Principally, this analysis leverages the CALSEC, a statewide population-based linkage, which provided us with a large sample size of cases and controls drawn from California birth records. This allowed us to examine the influence of PM_2.5_ exposure on a very rare cancer to better understand its etiology.** Because reporting of cancer diagnoses to cancer registries is required by law, and the CCR meets the high data standards for the National Program of Cancer Registries, we expect case ascertainment by the CCR to be near complete. There is still a possibility that a small number of cases might have been missed, but this should not pose a serious threat to the validity of our study, as Ewing sarcoma is extremely rare and the likelihood of any case being misclassified as a control in our study is close to zero**. Further, we controlled for multiple factors known to be associated with Ewing sarcoma risk in this population. Our registry-based study is unlikely to be affected by selection bias, as subjects are identified from registry records without being contacted for participation. Exposures were assigned while blinded to case/control status and were based on maternal residential address at the time of birth, which was documented before cancer diagnosis, eliminating recall bias.**

**There are several important limitations to this work.** For our exposure assessment, daily PM_2.5_ concentrations were only available from 2000-2016 and were extrapolated for children born prior to 2000. While the estimates produced by this model have been found to be highly concordant with measured PM_2.5_ concentrations in the Pacific region (R^2^ = 0.802),^56, 57^ it is possible that they do not accurately represent the true exposures incurred by these children, which could result in exposure misclassification**.^90^ Stratifying by births before and after 2000 was not possible due to the limited sample size. Residential mobility is a known source of exposure misclassification, particularly in studies of spatially varying exposures. Residential mobility has been associated with factors like SES and maternal age.^91, 92^ The exposure windows of interest were gestation and the first year after birth, which are relatively short in duration. While mothers of the cases and controls could have moved during this period, the impact likely had been moderate given the relatively short duration and our adjustment of individual- and community-level SES measures. Exposure misclassification from residential mobility is also typically thought to be nondifferential, which would bias the results towards the null. Further, o**ther studies of spatially defined environmental exposures have not found residential mobility to be a major source of error.^92, 93^ **Because we used a spatially based exposure modeling method, we excluded children who lacked sufficient maternal residential information. It is possible that this could introduce a non-differential selection mechanism which could lead to selection bias, but the bias would likely be nondifferential as the characteristics of the excluded cases and controls were similar. Finally, we are constrained to the information reported on the birth record or cancer registry data and lack additional individual-level information, such as diet or genetic ancestry. This could be a potential source of confounding if SES is collinear with genetic characteristics, particularly among the Hispanic children.**

Our results provide new suggestive evidence that perinatal exposure to ambient PM_2.5_ may contribute to Ewing sarcoma risk among Hispanic children in California, though there was no association in the combined population. These findings require replication and underscore the need to conduct further research in a persistently marginalized population who face a disproportionally heavier burden of environmental exposures. Future research should evaluate the potential modifying role of ethnicity in the PM_2.5_-cancer relationship to examine whether ethnicity is a proxy for or correlated with other unaccounted for social or structural factors, potentially incorporating genetic ancestry measures.

**Funding Declaration**

**CJC w**as supported by T32 training grants from the National Cancer Institute [T32CA250803 and T32CA099936]. The content of this manuscript is solely the responsibility of the authors and does not represent the official views of the National Institutes of Health.

**Acknowledgements**

We thank the Center for Health Statistics and Informatics within the California Department of Public Health for the birth data used in this analysis. The California Department of Public Health is not responsible for the analyses, interpretations, or conclusions drawn by the authors regarding the birth data used in this publication. The collection of cancer incidence data used in this study was supported by the California Department of Public Health pursuant to California Health and Safety Code Section 103885; Centers for Disease Control and Prevention’s National Program of Cancer Registries, under cooperative agreement 1NU58DP007156; the National Cancer Institute’s Surveillance, Epidemiology and End Results Program under contract HHSN261201800032I awarded to the University of California, San Francisco, contract HHSN261201800015I awarded to the University of Southern California, and contract HHSN261201800009I awarded to the Public Health Institute. The ideas and opinions expressed herein are those of the author(s) and do not necessarily reflect the opinions of the State of California, Department of Public Health, the National Cancer Institute, and the Centers for Disease Control and Prevention or their Contractors and Subcontractors.

**Supplemental Material for “Exposure to ambient fine particle air pollution during pregnancy and early life and risk of childhood Ewing sarcoma in a population-based case-control study in California (1988-2015)”**

Table S1. Ethnicity and maternal educational attainment stratified by age at diagnosis among Hispanic and non-Hispanic White case children.

| Age at Diagnosis | 0 to 4 (n=45) | 5 to 9 (n=65) | 10 to 14 (n=129) | 15 to 19 (n=117) |  |
| --- | --- | --- | --- | --- | --- |
|  | N (% of age group) | N (% of age group) | N (% of age group) | N (% of age group) | p-value |
| Ethnicity |  |  |  |  | 0.66 |
| Non-Hispanic White | 19 (42) | 35 (55) | 63 (49) | 60 (51) |  |
| Hispanic | 26 (58) | 30 (46) | 66 (51) | 57 (49) |  |
|  |  |  |  |  |  |
| Mother’s educational attainment |  |  |  |  | 0.09 |
| 8th grade or less | 4 (9) | 1 (1) | 12 (9) | 14 (12) |  |
| 9th - 12th grade | 24 (53) | 25 (38) | 40 (31) | 35 (30) |  |
| Some college or more | 15 (33) | 24 (37) | 46 (36) | 31 (27) |  |
| Unknown | 2 (4) | 15 (23) | 31 (24) | 37 (32) |  |

Table S2. Distribution of modeled PM_2.5_ concentrations (in µg/m^3^) among case and control children for the two considered exposure windows, stratified by Hispanic ethnicity.

|  | Non-Hispanic White (n= 6838*) | | Hispanic (n= 9577*) | |  |
| --- | --- | --- | --- | --- | --- |
| PM_2.5_ exposure (µg/m^3^) | Mean | Median (IQR) | Mean (SD) | Median (IQR) | p-value |
| Cases and Controls |  |  |  |  |  |
| Gestation | 21.60 (12.50) | 17.57 (12.84 - 26.89) | 24.63 (12.00) | 22.46 (15.08 - 31.45) | <0.01 |
| First Year After Birth | 20.90 (11.78) | 17.11 (12.68 - 25.91) | 23.74 (11.35) | 21.64 (14.72 - 30.14) | <0.01 |
|  |  |  |  |  |  |
| Case children only |  |  |  |  |  |
| Gestation | 20.62 (12.52) | 16.96 (12.30 - 25.33) | 25.13 (11.86) | 22.78 (16.22 - 31.53) | <0.01 |
| First Year After Birth | 20.00 (11.74) | 16.20 (12.36 - 24.22) | 24.23 (11.11) | 22.50 (15.65 - 30.28) | <0.01 |

*n = Combined number of case and control children. SD: Standard Deviation; IQR: Inter-Quartile Range.

Table S3. Associations between exposure to modeled ambient PM_2.5_ concentrations and Ewing sarcoma risk, including an indicator of a foreign-born mother, and excluding birth weight.

|  | Non-Hispanic White | Hispanic | Non-Hispanic White | Hispanic | Non-Hispanic White | Hispanic |
| --- | --- | --- | --- | --- | --- | --- |
| PM _2.5_ (µg/m^3^) | OR (95% CI) | OR (95% CI) | OR (95% CI) | OR (95% CI) | OR (95% CI) | OR (95% CI) |
|  |  |  |  |  |  |  |
| Gestational Period | Main model* | | Foreign mother** | | No birth weight*** | |
| Q1 (<13.68) | 1.00 | 1.00 | 1.00 | 1.00 | 1.00 | 1.00 |
| Q2 (13.68 - <20.08) | 0.74 (0.49, 1.10) | 1.53 (0.94, 2.51) | 0.74 (0.49, 1.10) | 1.52 (0.93, 2.50) | 0.74 (0.49, 1.10) | 1.52 (0.93, 2.48) |
| Q3 (20.08 - <29.90) | 0.80 (0.53, 1.23) | 1.56 (0.95, 2.56) | 0.80 (0.52, 1.23) | 1.56 (0.95, 2.5) | 0.80 (0.53, 1.23) | 1.56 (0.95, 2.56) |
| Q4 (≥29.90) | 0.66 (0.41, 1.07) | 1.39 (0.79, 2.47) | 0.66 (0.40, 1.07) | 1.41 (0.79, 2.50) | 0.66 (0.41, 1.07) | 1.38 (0.78, 2.45) |
|  |  |  |  |  |  |  |
| First Year After Birth |  |  |  |  |  |  |
| Q1 (<13.47) | 1.00 | 1.00 |  |  |  |  |
| Q2 (13.47 - <19.48) | 0.80 (0.54, 1.19) | 1.27 (0.77, 2.07) | 0.80 (0.54, 1.19) | 1.26 (0.77, 2.07) | 0.80 (0.54, 1.19) | 1.25 (0.77, 2.05) |
| Q3 (19.48 - <28.76) | 0.87 (0.57, 1.32) | 1.56 (0.96, 2.52) | 0.86 (0.57, 1.32) | 1.56 (0.97, 2.53) | 0.86 (0.57, 1.32) | 1.55 (0.96, 2.52) |
| Q4 (≥28.76) | 0.64 (0.39, 1.05) | 1.26 (0.71, 2.22) | 0.63 (0.38, 1.04) | 1.27 (0.72, 2.25) | 0.64 (0.39, 1.05) | 1.25 (0.71, 2.20) |
| * Adjusted for individual-level factors: birth year, sex, birth weight, and maternal educational attainment. | | | | | | |
| ** Adjusted for individual-level factors: birth year, sex, birth weight, maternal educational attainment, foreign-born mother. | | | | | | |
| *** Adjusted for individual-level factors: birth year, sex, and maternal educational attainment. | | | | | | |

Figure S1. Temporal trends in PM_2.5_ exposure among all case and control children during the study period (1982-2015), stratified by Hispanic ethnicity (Hispanic in red and non-Hispanic White in blue)


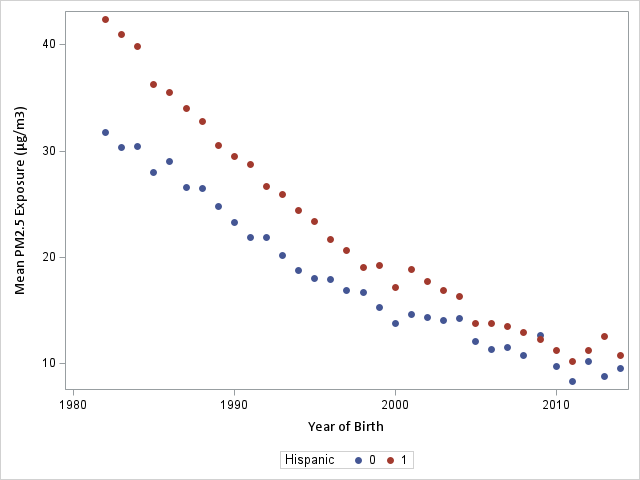


**References**

1. Jawad, M. U.; Cheung, M. C.; Min, E. S.; Schneiderbauer, M. M.; Koniaris, L. G.; Scully, S. P., Ewing sarcoma demonstrates racial disparities in incidence-related and sex-related differences in outcome: an analysis of 1631 cases from the SEER database, 1973-2005. *Cancer* **2009,** *115*, (15), 3526-36.

2. Grünewald, T. G. P.; Cidre-Aranaz, F.; Surdez, D.; Tomazou, E. M.; de Álava, E.; Kovar, H.; Sorensen, P. H.; Delattre, O.; Dirksen, U., Ewing sarcoma. *Nature Reviews Disease Primers* **2018,** *4*, (1), 5.

3. Potratz, J.; Dirksen, U.; Jürgens, H.; Craft, A., Ewing sarcoma: clinical state-of-the-art. *Pediatr Hematol Oncol* **2012,** *29*, (1), 1-11.

4. Hesla, A. C.; Papakonstantinou, A.; Tsagkozis, P., Current Status of Management and Outcome for Patients with Ewing Sarcoma. *Cancers (Basel)* **2021,** *13*, (6).

5. Gaspar, N.; Hawkins, D. S.; Dirksen, U.; Lewis, I. J.; Ferrari, S.; Le Deley, M. C.; Kovar, H.; Grimer, R.; Whelan, J.; Claude, L.; Delattre, O.; Paulussen, M.; Picci, P.; Sundby Hall, K.; van den Berg, H.; Ladenstein, R.; Michon, J.; Hjorth, L.; Judson, I.; Luksch, R.; Bernstein, M. L.; Marec-Bérard, P.; Brennan, B.; Craft, A. W.; Womer, R. B.; Juergens, H.; Oberlin, O., Ewing Sarcoma: Current Management and Future Approaches Through Collaboration. *J Clin Oncol* **2015,** *33*, (27), 3036-46.

6. Bhakta, N.; Liu, Q.; Ness, K. K.; Baassiri, M.; Eissa, H.; Yeo, F.; Chemaitilly, W.; Ehrhardt, M. J.; Bass, J.; Bishop, M. W.; Shelton, K.; Lu, L.; Huang, S.; Li, Z.; Caron, E.; Lanctot, J.; Howell, C.; Folse, T.; Joshi, V.; Green, D. M.; Mulrooney, D. A.; Armstrong, G. T.; Krull, K. R.; Brinkman, T. M.; Khan, R. B.; Srivastava, D. K.; Hudson, M. M.; Yasui, Y.; Robison, L. L., The cumulative burden of surviving childhood cancer: an initial report from the St Jude Lifetime Cohort Study (SJLIFE). *Lancet* **2017,** *390*, (10112), 2569-2582.

7. Oeffinger, K. C.; Mertens, A. C.; Sklar, C. A.; Kawashima, T.; Hudson, M. M.; Meadows, A. T.; Friedman, D. L.; Marina, N.; Hobbie, W.; Kadan-Lottick, N. S.; Schwartz, C. L.; Leisenring, W.; Robison, L. L., Chronic health conditions in adult survivors of childhood cancer. *N Engl J Med* **2006,** *355*, (15), 1572-82.

8. Mertens, A. C.; Liu, Q.; Neglia, J. P.; Wasilewski, K.; Leisenring, W.; Armstrong, G. T.; Robison, L. L.; Yasui, Y., Cause-specific late mortality among 5-year survivors of childhood cancer: the Childhood Cancer Survivor Study. *J Natl Cancer Inst* **2008,** *100*, (19), 1368-79.

9. Hudson, M. M.; Mertens, A. C.; Yasui, Y.; Hobbie, W.; Chen, H.; Gurney, J. G.; Yeazel, M.; Recklitis, C. J.; Marina, N.; Robison, L. R.; Oeffinger, K. C.; Investigators, f. t. C. C. S. S., Health Status of Adult Long-term Survivors of Childhood CancerA Report From the Childhood Cancer Survivor Study. *JAMA* **2003,** *290*, (12), 1583-1592.

10. Baytan, B.; Aşut, Ç.; Çırpan Kantarcıoğlu, A.; Sezgin Evim, M.; Güneş, A. M., Health-Related Quality of Life, Depression, Anxiety, and Self-Image in Acute Lymphocytic Leukemia Survivors. *Turkish journal of haematology : official journal of Turkish Society of Haematology* **2016,** *33*, (4), 326-330.

11. Ginsberg, J. P.; Goodman, P.; Leisenring, W.; Ness, K. K.; Meyers, P. A.; Wolden, S. L.; Smith, S. M.; Stovall, M.; Hammond, S.; Robison, L. L.; Oeffinger, K. C., Long-term Survivors of Childhood Ewing Sarcoma: Report From the Childhood Cancer Survivor Study. *JNCI: Journal of the National Cancer Institute* **2010,** *102*, (16), 1272-1283.

12. Spector, L. G.; Hubbard, A. K.; Diessner, B. J.; Machiela, M. J.; Webber, B. R.; Schiffman, J. D., Comparative international incidence of Ewing sarcoma 1988 to 2012. *International Journal of Cancer* **2021,** *149*, (5), 1054-1066.

13. Dupuy, M.; Lamoureux, F.; Mullard, M.; Postec, A.; Regnier, L.; Baud’huin, M.; Georges, S.; Brounais-Le Royer, B.; Ory, B.; Rédini, F.; Verrecchia, F., Ewing sarcoma from molecular biology to the clinic. *Frontiers in Cell and Developmental Biology* **2023,** *11*.

14. Helman, L. J.; Meltzer, P., Mechanisms of sarcoma development. *Nature Reviews Cancer* **2003,** *3*, (9), 685-694.

15. Riggi, N.; Stamenkovic, I., The Biology of Ewing sarcoma. *Cancer Letters* **2007,** *254*, (1), 1-10.

16. Ohno, T.; Ouchida, M.; Lee, L.; Gatalica, Z.; Rao, V. N.; Reddy, E. S., The EWS gene, involved in Ewing family of tumors, malignant melanoma of soft parts and desmoplastic small round cell tumors, codes for an RNA binding protein with novel regulatory domains. *Oncogene* **1994,** *9*, (10), 3087-3097.

17. Pereira, R.; Quang, C. T.; Lesault, I.; Dolznig, H.; Beug, H.; Ghysdael, J., FLI-1 inhibits differentiation and induces proliferation of primary erythroblasts. *Oncogene* **1999,** *18*, (8), 1597-1608.

18. Lin, P. P.; Wang, Y.; Lozano, G., Mesenchymal Stem Cells and the Origin of Ewing's Sarcoma. *Sarcoma* **2011,** *2011*, 276463.

19. Ross, K. A.; Smyth, N. A.; Murawski, C. D.; Kennedy, J. G., The Biology of Ewing Sarcoma. *ISRN Oncology* **2013,** *2013*, 759725.

20. Funes, J. M.; Quintero, M.; Henderson, S.; Martinez, D.; Qureshi, U.; Westwood, C.; Clements, M. O.; Bourboulia, D.; Pedley, R. B.; Moncada, S.; Boshoff, C., Transformation of human mesenchymal stem cells increases their dependency on oxidative phosphorylation for energy production. *Proceedings of the National Academy of Sciences* **2007,** *104*, (15), 6223-6228.

21. Riggi, N.; Cironi, L.; Provero, P.; Suvà, M. L.; Kaloulis, K.; Garcia-Echeverria, C.; Hoffmann, F.; Trumpp, A.; Stamenkovic, I., Development of Ewing's sarcoma from primary bone marrow-derived mesenchymal progenitor cells. *Cancer Res* **2005,** *65*, (24), 11459-68.

22. Kovar, H.; Amatruda, J.; Brunet, E.; Burdach, S.; Cidre-Aranaz, F.; de Alava, E.; Dirksen, U.; van der Ent, W.; Grohar, P.; Grünewald, T. G.; Helman, L.; Houghton, P.; Iljin, K.; Korsching, E.; Ladanyi, M.; Lawlor, E.; Lessnick, S.; Ludwig, J.; Meltzer, P.; Metzler, M.; Mora, J.; Moriggl, R.; Nakamura, T.; Papamarkou, T.; Radic Sarikas, B.; Rédini, F.; Richter, G. H.; Rossig, C.; Schadler, K.; Schäfer, B. W.; Scotlandi, K.; Sheffield, N. C.; Shelat, A.; Snaar-Jagalska, E.; Sorensen, P.; Stegmaier, K.; Stewart, E.; Sweet-Cordero, A.; Szuhai, K.; Tirado, O. M.; Tirode, F.; Toretsky, J.; Tsafou, K.; Üren, A.; Zinovyev, A.; Delattre, O., The second European interdisciplinary Ewing sarcoma research summit--A joint effort to deconstructing the multiple layers of a complex disease. *Oncotarget* **2016,** *7*, (8), 8613-24.

23. Valery, P. C.; McWhirter, W.; Sleigh, A.; Williams, G.; Bain, C., Farm exposures, parental occupation, and risk of Ewing's sarcoma in Australia: a national case–control study. *Cancer Causes & Control* **2002,** *13*, (3), 263-270.

24. Vinson, F.; Merhi, M.; Baldi, I.; Raynal, H.; Gamet-Payrastre, L., Exposure to pesticides and risk of childhood cancer: a meta-analysis of recent epidemiological studies. *Occupational and Environmental Medicine* **2011,** *68*, (9), 694-702.

25. Infante-Rivard, C.; Weichenthal, S., Pesticides and childhood cancer: an update of Zahm and Ward's 1998 review. *J Toxicol Environ Health B Crit Rev* **2007,** *10*, (1-2), 81-99.

26. Zahm, S. H.; Ward, M. H., Pesticides and childhood cancer. *Environmental Health Perspectives* **1998,** *106*, (suppl 3), 893-908.

27. Holly, E. A.; Aston, D. A.; Ahn, D. K.; Kristiansen, J. J., Ewing's Bone Sarcoma, Paternal Occupational Exposure, and Other Factors. *American Journal of Epidemiology* **1992,** *135*, (2), 122-129.

28. Moore, L. E.; Gold, L.; Stewart, P. A.; Gridley, G.; Prince, J. R.; Zahm, S. H., Parental occupational exposures and Ewing's sarcoma. *Int J Cancer* **2005,** *114*, (3), 472-8.

29. García-Pérez, J.; Morales-Piga, A.; Gómez-Barroso, D.; Tamayo-Uria, I.; Romaguera, E. P.; López-Abente, G.; Ramis, R., Risk of bone tumors in children and residential proximity to industrial and urban areas: New findings from a case-control study. *The Science of The Total Environment* **2017,** *579*, 1333-1342.

30. Frazier, R., State investigating potential cancer cluster in Washington County; some residents fear an environmental cause. *StateImpact Pennsylvania* March 25, 2019.

31. Langley, R., Hirsch, A., Ewing Sarcoma Investigation in Wake County, North Carolina. In North Caroline Department of Health and Human Servies Division of Public Health, Ed. 2013.

32. Viel, J.-F.; Arveux, P.; Baverel, J.; Cahn, J.-Y., Soft-Tissue Sarcoma and Non-Hodgkin's Lymphoma Clusters around a Municipal Solid Waste Incinerator with High Dioxin Emission Levels. *American Journal of Epidemiology* **2000,** *152*, (1), 13-19.

33. Kelly, F. J., Oxidative stress: its role in air pollution and adverse health effects. *Occup Environ Med* **2003,** *60*, (8), 612-6.

34. Møller, P.; Danielsen, P. H.; Karottki, D. G.; Jantzen, K.; Roursgaard, M.; Klingberg, H.; Jensen, D. M.; Christophersen, D. V.; Hemmingsen, J. G.; Cao, Y.; Loft, S., Oxidative stress and inflammation generated DNA damage by exposure to air pollution particles. *Mutat Res Rev Mutat Res* **2014,** *762*, 133-66.

35. Vadillo-Ortega, F.; Osornio-Vargas, A.; Buxton, M. A.; Sánchez, B. N.; Rojas-Bracho, L.; Viveros-Alcaráz, M.; Castillo-Castrejón, M.; Beltrán-Montoya, J.; Brown, D. G.; O'Neill, M. S., Air pollution, inflammation and preterm birth: a potential mechanistic link. *Med Hypotheses* **2014,** *82*, (2), 219-24.

36. Benbrahim-Tallaa, L.; Lauby-Secretan, B.; Loomis, D.; Guyton, K. Z.; Grosse, Y.; El Ghissassi, F.; Bouvard, V.; Guha, N.; Mattock, H.; Straif, K., Carcinogenicity of perfluorooctanoic acid, tetrafluoroethylene, dichloromethane, 1, 2-dichloropropane, and 1, 3-propane sultone. *Lancet Oncology* **2014,** *15*, (9), 924.

37. Ghosh, J. K. C.; Heck, J. E.; Cockburn, M.; Su, J.; Jerrett, M.; Ritz, B., Prenatal Exposure to Traffic-related Air Pollution and Risk of Early Childhood Cancers. *American Journal of Epidemiology* **2013,** *178*, (8), 1233-1239.

38. Raaschou-Nielsen, O.; Reynolds, P., Air pollution and childhood cancer: A review of the epidemiological literature. *International Journal of Cancer* **2006,** *118*, (12), 2920-2929.

39. Filippini, T.; Heck, J. E.; Malagoli, C.; Giovane, C. D.; Vinceti, M., A Review and Meta-Analysis of Outdoor Air Pollution and Risk of Childhood Leukemia. *Journal of Environmental Science and Health, Part C* **2015,** *33*, (1), 36-66.

40. Buffler, P. A.; Kwan, M. L.; Reynolds, P.; Urayama, K. Y., Environmental and genetic risk factors for childhood leukemia: appraising the evidence. *Cancer Invest* **2005,** *23*, (1), 60-75.

41. Heck Julia, E.; Wu, J.; Lombardi, C.; Qiu, J.; Meyers Travis, J.; Wilhelm, M.; Cockburn, M.; Ritz, B., Childhood Cancer and Traffic-Related Air Pollution Exposure in Pregnancy and Early Life. *Environmental Health Perspectives* **2013,** *121*, (11-12), 1385-1391.

42. Hvidtfeldt, U. A.; Erdmann, F.; Urhoj, S. K.; Brandt, J.; Geels, C.; Ketzel, M.; Frohn, L. M.; Christensen, J. H.; Sørensen, M.; Raaschou-Nielsen, O., Residential Exposure to PM2.5 Components and Risk of Childhood Non-Hodgkin Lymphoma in Denmark: A Nationwide Register-Based Case-Control Study. *International Journal of Environmental Research and Public Health* **2020,** *17*, (23), 8949.

43. Lee, J. M.; Lee, T.-H.; Kim, S.; Song, M.; Bae, S., Association between long-term exposure to particulate matter and childhood cancer: A retrospective cohort study. *Environmental Research* **2022,** *205*, 112418.

44. Williams, L. A.; Haynes, D.; Sample, J. M.; Lu, Z.; Hossaini, A.; McGuinn, L. A.; Hoang, T. T.; Lupo, P. J.; Scheurer, M. E., PM2.5, vegetation density, and childhood cancer: a case-control registry-based study from Texas 1995-2011. *JNCI: Journal of the National Cancer Institute* **2024**.

45. Ghosh, J. K.; Heck, J. E.; Cockburn, M.; Su, J.; Jerrett, M.; Ritz, B., Prenatal exposure to traffic-related air pollution and risk of early childhood cancers. *Am J Epidemiol* **2013,** *178*, (8), 1233-9.

46. Morello-Frosch, R.; Pastor, M.; Sadd, J., Environmental Justice and Southern California’s “Riskscape”: The Distribution of Air Toxics Exposures and Health Risks among Diverse Communities. *Urban Affairs Review* **2001,** *36*, (4), 551-578.

47. Bullard, R. D.; Wright, B. H., Environmental Justice for all: Community Perspectives on Health and Research. *Toxicology and Industrial Health* **1993,** *9*, (5), 821-841.

48. Liu, J.; Clark, L. P.; Bechle, M. J.; Hajat, A.; Kim, S.-Y.; Robinson, A. L.; Sheppard, L.; Szpiro, A. A.; Marshall, J. D., Disparities in Air Pollution Exposure in the United States by Race/Ethnicity and Income, 1990–2010. *Environmental Health Perspectives* **2021,** *129*, (12), 127005.

49. Perlin, S. A.; Wong, D.; Sexton, K., Residential Proximity to Industrial Sources of Air Pollution: Interrelationships among Race, Poverty, and Age. *Journal of the Air & Waste Management Association* **2001,** *51*, (3), 406-421.

50. Marcotte, E. L.; Domingues, A. M.; Sample, J. M.; Richardson, M. R.; Spector, L. G., Racial and ethnic disparities in pediatric cancer incidence among children and young adults in the United States by single year of age. *Cancer* **2021,** *127*, (19), 3651-3663.

51. Rice, A. K.; McCray, J. E.; Singha, K., Numerical Investigation of Wellbore Methane Leakage From a Dual-Porosity Reservoir and Subsequent Transport in Groundwater. **2021,** *57*, (2), e2019WR026991.

52. Bell, M. L.; Dominici, F.; Ebisu, K.; Zeger, S. L.; Samet, J. M., Spatial and Temporal Variation in PM<sub>2.5</sub> Chemical Composition in the United States for Health Effects Studies. *Environmental Health Perspectives* **2007,** *115*, (7), 989-995.

53. Wiemels, J. L.; Cazzaniga, G.; Daniotti, M.; Eden, O. B.; Addison, G. M.; Masera, G.; Saha, V.; Biondi, A.; Greaves, M. F., Prenatal origin of acute lymphoblastic leukaemia in children. *Lancet* **1999,** *354*, (9189), 1499-503.

54. Hajat, A.; MacLehose, R. F.; Rosofsky, A.; Walker, K. D.; Clougherty, J. E., Confounding by Socioeconomic Status in Epidemiological Studies of Air Pollution and Health: Challenges and Opportunities. *Environmental Health Perspectives* **2021,** *129*, (6), 065001.

55. Agency for Toxic Substances and Disease Registry (ATSDR) The social vulnerability index. <https://www.atsdr.cdc.gov/placeandhealth/svi/index.html>

56. Di, Q.; Amini, H.; Shi, L.; Kloog, I.; Silvern, R.; Kelly, J.; Sabath, M. B.; Choirat, C.; Koutrakis, P.; Lyapustin, A., An ensemble-based model of PM2. 5 concentration across the contiguous United States with high spatiotemporal resolution. *Environment international* **2019,** *130*, 104909.

57. Di, Q.; Kloog, I.; Koutrakis, P.; Lyapustin, A.; Wang, Y.; Schwartz, J., Assessing PM2. 5 exposures with high spatiotemporal resolution across the continental United States. *Environmental science & technology* **2016,** *50*, (9), 4712-4721.

58. Zhong, C.; Wang, R.; Morimoto, L. M.; Longcore, T.; Franklin, M.; Rogne, T.; Metayer, C.; Wiemels, J. L.; Ma, X., Outdoor artificial light at night, air pollution, and risk of childhood acute lymphoblastic leukemia in the California Linkage Study of Early-Onset Cancers. *Sci Rep* **2023,** *13*, (1), 583.

59. Selevan, S. G.; Kimmel, C. A.; Mendola, P., Identifying critical windows of exposure for children's health. *Environmental Health Perspectives* **2000,** *108*, (suppl 3), 451-455.

60. Olshan, A. F.; Anderson, L.; Roman, E.; Fear, N.; Wolff, M.; Whyatt, R.; Vu, V.; Diwan, B. A.; Potischman, N., Workshop to identify critical windows of exposure for children's health: cancer work group summary. *Environmental Health Perspectives* **2000,** *108*, (suppl 3), 595-597.

61. Vodonos, A.; Awad, Y. A.; Schwartz, J., The concentration-response between long-term PM2.5 exposure and mortality; A meta-regression approach. *Environmental Research* **2018,** *166*, 677-689.

62. Daniels, M. J.; Dominici, F.; Samet, J. M.; Zeger, S. L., Estimating particulate matter-mortality dose-response curves and threshold levels: an analysis of daily time-series for the 20 largest US cities. *Am J Epidemiol* **2000,** *152*, (5), 397-406.

63. Schwartz, J.; Laden, F.; Zanobetti, A., The concentration-response relation between PM(2.5) and daily deaths. *Environ Health Perspect* **2002,** *110*, (10), 1025-9.

64. Wiemels, J. L.; Wang, R.; Feng, Q.; Yee, A. C.; Morimoto, L. M.; Metayer, C.; Ma, X., Birth characteristics and risk of Ewing sarcoma. *Cancer Causes & Control* **2023,** *34*, (10), 837-843.

65. Mohai, P.; Bryant, B., Race, Poverty & the Distribution of Environmental Hazards: Reviewing the Evidence. *Race, Poverty & the Environment* **1991,** *2*, (3/4), 3-27.

66. Collins, M. B.; Munoz, I.; JaJa, J., Linking ‘toxic outliers’ to environmental justice communities. *Environmental Research Letters* **2016,** *11*, (1), 015004.

67. Banzhaf, S.; Ma, L.; Timmins, C., Environmental Justice: The Economics of Race, Place, and Pollution. *Journal of Economic Perspectives* **2019,** *33*, (1), 185-208.

68. Wolch, J. R.; Byrne, J.; Newell, J. P., Urban green space, public health, and environmental justice: The challenge of making cities ‘just green enough’. *Landscape and Urban Planning* **2014,** *125*, 234-244.

69. Byrne, J., When green is White: The cultural politics of race, nature and social exclusion in a Los Angeles urban national park. *Geoforum* **2012,** *43*, (3), 595-611.

70. Bullard, R. D., Solid Waste Sites and the Black Houston Community*. *Sociological Inquiry* **1983,** *53*, (2‐3), 273-288.

71. Pastor, M.; Sadd, J.; Hipp, J., Which Came First? Toxic Facilities, Minority Move-In, and Environmental Justice. *Journal of Urban Affairs* **2001,** *23*, (1), 1-21.

72. Switzer, D.; Teodoro, M. P., Class, Race, Ethnicity, and Justice in Safe Drinking Water Compliance*. *Social Science Quarterly* **2018,** *99*, (2), 524-535.

73. Balazs, C.; Morello-Frosch, R.; Hubbard, A.; Ray, I., Social disparities in nitrate-contaminated drinking water in California's San Joaquin Valley. *Environmental Health Perspectives* **2011,** *119*, (9), 1272-1278.

74. Schaider, L. A.; Swetschinski, L.; Campbell, C.; Rudel, R. A., Environmental justice and drinking water quality: Are there socioeconomic disparities in nitrate levels in U.S. drinking water? *Environmental Health: A Global Access Science Source* **2019,** *18*, (1).

75. Heck, J. E.; Park, A. S.; Contreras, Z. A.; Davidson, T. B.; Hoggatt, K. J.; Cockburn, M.; Ritz, B., Risk of Childhood Cancer by Maternal Birthplace: A Test of the Hispanic Paradox. *JAMA Pediatrics* **2016,** *170*, (6), 585-592.

76. Feero, W. G.; Steiner, R. D.; Slavotinek, A.; Faial, T.; Bamshad, M. J.; Austin, J.; Korf, B. R.; Flanagin, A.; Bibbins-Domingo, K., Guidance on Use of Race, Ethnicity, and Geographic Origin as Proxies for Genetic Ancestry Groups in Biomedical Publications. *Jama* **2024,** *331*, (15), 1276-1278.

77. Hong, H. L.; Ton, T. V.; Devereux, T. R.; Moomaw, C.; Clayton, N.; Chan, P.; Dunnick, J. K.; Sills, R. C., Chemical-specific alterations in ras, p53, and β-catenin genes in hemangiosarcomas from B6C3F1 mice exposed to o-nitrotoluene or riddelliine for 2 years. *Toxicology and Applied Pharmacology* **2003,** *191*, (3), 227-234.

78. Rhodes, M. C.; Bucher, J. R.; Peckham, J. C.; Kissling, G. E.; Hejtmancik, M. R.; Chhabra, R. S., Carcinogenesis studies of benzophenone in rats and mice. *Food and Chemical Toxicology* **2007,** *45*, (5), 843-851.

79. Huang, W.-Y.; Chen, Y.-F.; Huang, K.-Y., The association between ambient air pollution exposure and connective tissue sarcoma risk: a nested case–control study using a nationwide population-based database. *Environmental Science and Pollution Research* **2024,** *31*, (6), 9078-9090.

80. Zambon, P.; Ricci, P.; Bovo, E.; Casula, A.; Gattolin, M.; Fiore, A. R.; Chiosi, F.; Guzzinati, S., Sarcoma risk and dioxin emissions from incinerators and industrial plants: a population-based case-control study (Italy). *Environmental Health* **2007,** *6*, (1), 19.

81. U.S. Environmental Protection Agency, Reconsideration of the National Ambient Air Quality Standards for Particulate Matter. In 2024; Vol. 89 FR 16202, pp 16202-16406.

82. Park, A. S.; Ritz, B.; Ling, C.; Cockburn, M.; Heck, J. E., Exposure to ambient dichloromethane in pregnancy and infancy from industrial sources and childhood cancers in California. *International Journal of Hygiene and Environmental Health* **2017,** *220*, (7), 1133-1140.

83. Heck, J. E.; He, D.; Wing, S. E.; Ritz, B.; Carey, C. D.; Yang, J.; Stram, D. O.; Le Marchand, L.; Park, S. L.; Cheng, I.; Wu, A. H., Exposure to outdoor ambient air toxics and risk of breast cancer: The multiethnic cohort. *International Journal of Hygiene and Environmental Health* **2024,** *259*, 114362.

84. Colmer, J.; Hardman, I.; Shimshack, J.; Voorheis, J., Disparities in PM<sub>2.5</sub> air pollution in the United States. *Science* **2020,** *369*, (6503), 575-578.

85. Chambliss, S. E.; Pinon, C. P. R.; Messier, K. P.; LaFranchi, B.; Upperman, C. R.; Lunden, M. M.; Robinson, A. L.; Marshall, J. D.; Apte, J. S., Local- and regional-scale racial and ethnic disparities in air pollution determined by long-term mobile monitoring. *Proceedings of the National Academy of Sciences* **2021,** *118*, (37), e2109249118.

86. Carozza, S. E.; Puumala, S. E.; Chow, E. J.; Fox, E. E.; Horel, S.; Johnson, K. J.; McLaughlin, C. C.; Reynolds, P.; Von Behren, J.; Mueller, B. A.; Spector, L. G., Parental educational attainment as an indicator of socioeconomic status and risk of childhood cancers. *British Journal of Cancer* **2010,** *103*, (1), 136-142.

87. Diessner, B. J.; Weigel, B. J.; Murugan, P.; Zhang, L.; Poynter, J. N.; Spector, L. G., Racial and Ethnic Differences in Sarcoma Incidence Are Independent of Census-Tract Socioeconomic Status. *Cancer Epidemiology, Biomarkers & Prevention* **2020,** *29*, (11), 2141-2148.

88. Braveman, P. A.; Cubbin, C.; Egerter, S.; Chideya, S.; Marchi, K. S.; Metzler, M.; Posner, S., Socioeconomic status in health research: one size does not fit all. *Jama* **2005,** *294*, (22), 2879-88.

89. Florez, J. C.; Price, A. L.; Campbell, D.; Riba, L.; Parra, M. V.; Yu, F.; Duque, C.; Saxena, R.; Gallego, N.; Tello-Ruiz, M.; Franco, L.; Rodríguez-Torres, M.; Villegas, A.; Bedoya, G.; Aguilar-Salinas, C. A.; Tusié-Luna, M. T.; Ruiz-Linares, A.; Reich, D., Strong association of socioeconomic status with genetic ancestry in Latinos: implications for admixture studies of type 2 diabetes. *Diabetologia* **2009,** *52*, (8), 1528-36.

90. Kim, S. Y.; Olives, C.; Sheppard, L.; Sampson, P. D.; Larson, T. V.; Keller, J. P.; Kaufman, J. D., Historical Prediction Modeling Approach for Estimating Long-Term Concentrations of PM2.5 in Cohort Studies before the 1999 Implementation of Widespread Monitoring. *Environ Health Perspect* **2017,** *125*, (1), 38-46.

91. Fell, D. B.; Dodds, L.; King, W. D., Residential mobility during pregnancy. *Paediatric and Perinatal Epidemiology* **2004,** *18*, (6), 408-414.

92. Bell, M. L.; Belanger, K., Review of research on residential mobility during pregnancy: consequences for assessment of prenatal environmental exposures. *J Expo Sci Environ Epidemiol* **2012,** *22*, (5), 429-38.

93. Bell, M. L.; Banerjee, G.; Pereira, G., Residential mobility of pregnant women and implications for assessment of spatially-varying environmental exposures. *J Expo Sci Environ Epidemiol* **2018,** *28*, (5), 470-480.
